# Postoperative Opioid Administration and Prescription Practices Following Hysterectomy in Two Tertiary Care Centres: A Comparative Cohort Study between Canada and Austria

**DOI:** 10.3390/jcm13206031

**Published:** 2024-10-10

**Authors:** Judith Schiefer, Julian Marschalek, Djurdjica Djuric, Samantha Benlolo, Eliane M. Shore, Guylaine Lefebvre, Lorenz Kuessel, Christof Worda, Heinrich Husslein

**Affiliations:** 1 Medical University of Vienna, Department of Anaesthesia, Intensive Care Medicine and Pain Medicine, Division of General Anaesthesia and Intensive Care Medicine, 1090 Vienna, Austria; judith.schiefer@meduniwien.ac.at; 2 Medical University of Vienna, Department of Obstetrics and Gynaecology, 1090 Vienna, Austria; julian.marschalek@meduniwien.ac.at (J.M.); n01442464@students.meduniwien.ac.at (D.D.); lorenz.kuessel@meduniwien.ac.at (L.K.); christof.worda@meduniwien.ac.at (C.W.); 3 Department of Obstetrics and Gynaecology, St. Michael’s Hospital, Toronto, ON M5C 2T2, Canada; samantha.benlolo@gmail.com (S.B.); eliane.shore@unityhealth.to (E.M.S.); guylainetmarc@me.com (G.L.)

**Keywords:** benign hysterectomy, geographic variation, opioid prescription, pain management, pain treatment

## Abstract

**Background:** In light of the opioid epidemic, opioid-prescribing modalities for postoperative pain management have been discussed controversially and show a wide variation across geographic regions. The aim of this study was to compare postoperative pain treatment regimes. **Methods:** We performed a matched cohort study of women undergoing hysterectomy in Austria (*n* = 200) and Canada (*n* = 200). We aimed to compare perioperative opioid medications, converted to morphine equivalent dose (MED) and doses of non-opioid analgesic (NOA) within the first 24 h after hysterectomy, and opioid prescriptions at discharge between the two cohorts. **Results:** The total MED received intraoperatively, in the post-anaesthesia care unit (PACU) and during the first 24 h after surgery, was similar in both cohorts (145.59 vs. 137.87; *p* = 0.17). Women in the Austrian cohort received a higher MED intraoperatively compared to the Canadian cohort (117.24 vs. 79.62; *p* < 0.001) but a lower MED in the PACU (25.96 vs. 30.42; *p* = 0.04). The primary outcome, MED within 24 h in the postoperative ward, was markedly lower in the Austrian compared to the Canadian cohort (2.36 vs. 27.98; *p* < 0.001). In a regression analysis, only the variables “Country” and “mode of hysterectomy” affected this outcome. A total of 98.5% in the Canadian cohort were given an opioid prescription at discharge vs. 0% in the Austrian cohort. **Conclusions:** Our analysis reveals marked differences between Austria and Canada regarding pain management practices following elective hysterectomy; the significantly higher intraoperative and significantly lower postoperative MED administration in the Austrian cohort compared to the Canadian cohort seems to be significantly affected by each country’s cultural attitudes towards pain management; this may have significant public health consequences and warrants further research.

## 1. Introduction

The opioid epidemic in North America has led to a number of efforts to reduce the risk of addiction and/or abuse of these prescription medicines [1,2]. Chronic opioid use/abuse places a substantial burden on both the afflicted person’s health and the socioeconomic system [1,3].

Elective surgery is a potential risk factor for chronic opioid use in previously opioid-naïve patients [4,5,6]. Hysterectomy is one of the most frequently performed elective gynaecologic surgeries. Adequate pain management is a critical component in achieving prompt postsurgical mobilization, as well as expediting recovery and hospital discharge; therefore, in many countries, prescription opioids have become common practice for managing postoperative pain [7]. However, receiving opioids perioperatively, receiving an opioid prescription at discharge, and the duration of opioid usage have all been proposed to be strong risk factors for developing an opioid use disorder [8,9,10]. Further, the US Centres for Disease Control and Prevention reported liberal opioid-prescribing practices as a public health concern, with 35% of all opioid overdose-related deaths in 2017 being directly attributed to opioid prescriptions [11]. In the US, surgeons write up to 36% of all opioid prescriptions [12], hence facing a challenging task: to provide their patients with adequate postoperative pain management while avoiding overprescribing opioids. Moreover, a 2022 review focusing on opioid vs. opioid-free analgesia after surgical discharge from minor or moderate–complex elective surgery indicated that a prescription of opioids at surgical discharge does not alleviate pain intensity but is associated with an increased risk of adverse events [13].

Despite this well-recognized public health concern, we still lack international consensus regarding opioid-prescribing practices, resulting in wide geographical variations. Some countries—particularly in North America—take a liberal approach to prescribing opioids, whereas many European countries such as France, Germany, and Austria are more restrictive [14,15].

Here, we compared postoperative pain management modalities between two tertiary care centres in Austria and Canada following hysterectomy.

## 2. Materials and Methods

We performed a matched cohort study involving a prospective cohort of 200 women undergoing hysterectomy for a benign indication at St. Michael’s Hospital in Toronto, Canada (affiliated with the University of Toronto), and a retrospective cohort of 200 women who underwent hysterectomy for a benign indication at the General Hospital of Vienna in Vienna, Austria (affiliated with the Medical University of Vienna), according to the Canadian Task Force Classification II-2. Both of these sites are large academic medical centres with trainees involved in the surgical procedures and postoperative care of patients.

The study was approved by the local ethical review boards of St. Michael’s Hospital, Toronto, Canada (13-238; date of approval: 11 October 2013), and the Medical University of Vienna, Austria (EK 2348/2019; date of approval: 12 February 2020), and was performed in accordance with the Declaration of Helsinki and the guidelines of Good Scientific Practice. Informed consent (both oral and written) was obtained from all participants in the prospective Canadian cohort.

### 2.1. Prospective Canadian Cohort

From November 2013 through November 2014, patients scheduled to undergo hysterectomy for a benign or select malignant indication were invited to participate in a study that aimed to predict persistent postsurgical pain following hysterectomy, the results of which were published previously, including a detailed description of the study design and the parameters collected [16]. In short, the inclusion criteria were as follows: age ≥ 18 years, sufficient knowledge of the English language to provide consent and respond to questionnaires, and hysterectomy performed by any route (laparotomy, laparoscopy, vaginal, or robotic) under general anaesthesia either with or without an additional procedure. The exclusion criteria included the following: malignancy (with the exception of low-grade endometrial cancer) and emergency hysterectomy. Participants completed preoperative questionnaires to evaluate their baseline pain levels and psychological factors. Pain assessments were conducted at both 1 and 24 h after surgery. Additionally, patients were re-evaluated six weeks post-surgery.

### 2.2. Retrospective Austrian Cohort

This cohort included retrospectively collected data from all women who underwent hysterectomy under general anaesthesia for a benign indication from January 2013 through December 2016. The patients in this cohort were matched to the Canadian cohort with respect to age, BMI, and surgical route, and the inclusion and exclusion criteria were identical to the criteria described above for the prospective Canadian cohort.

### 2.3. Parameters

Patient demographics, including body mass index (BMI), American Society of Anesthesiologists (ASA) score, parity, menopausal status, and smoking status, were recorded for both cohorts. In addition, we obtained information regarding the surgery, including the indication for surgery, the surgical approach (i.e., laparotomy, laparoscopy, vaginal, robotic), additional surgical procedures performed during the hysterectomy, the length of surgery, and the total length of hospital stay.

Directly before transfer from the post-anaesthesia care unit (PACU) to the postoperative ward, each patient’s level of pain was assessed using a visual analogue scale (VAS).

We also noted all intraoperative opioid and non-opioid analgesic (NOA) medications administered, preoperative and intraoperative use of benzodiazepines and co-analgesics, opioid and NOA medications administered within 24 h after surgery, and whether the patient received an opioid prescription at the time of discharge for managing postoperative pain. Patients received their prescribed pain medication on the ward either as part of a fixed regimen or pro re nata (PRN), depending on the surgeon’s policy, and additional doses were given as prescribed if pain was scored as 4 or higher on the VAS.

To facilitate our analysis, we converted all opioid medications to a morphine equivalent dose (MED), as described previously [17]. NOAs were converted to “number of doses” using the equivalent of a standard dose of the respective NOA (e.g., 1 g paracetamol, 75 mg diclofenac, and 15 mg ketorolac were each considered one dose). Doses of preoperative and intraoperative co-analgesics (e.g., clonidine, ketamine, local anaesthetics, and glucocorticoids) were converted using a similar approach.

The study’s primary endpoint was to compare MED administered within 24 h in the postoperative ward after hysterectomy between the Austrian and Canadian cohorts and define the variables that affect this outcome. As secondary outcomes, intraoperative MED, the level of pain before transfer from the PACU, MED in the PACU, and total MED (i.e., intraoperatively, in the PACU, and within the first 24 h in the postoperative ward) were compared, as well as whether patients received an opioid prescription at the time of discharge.

### 2.4. Statistical Analysis

Statistical analyses were performed using SPSS version 25 for Mac OS X (IBM SPSS Inc., Armonk, NY, USA). Continuous variables are reported as the mean ± standard deviation, and groups were compared using the Student’s *t*-test or the Mann–Whitney *U* test. Categorical variables were analysed using the Kruskal–Wallis H test, Chi-square test, or Fisher’s exact test. To analyse the relationship between different variables, we used a multivariate generalized linear model with a linear scale response and report the regression coefficient (Beta), standard error of the regression coefficient, β, and 95% confidence interval. Differences were considered statistically significant at *p* ≤ 0.05.

## 3. Results

A total of 400 women (200 each in the AUT and CAN cohorts) were included in this study; the patient characteristics are summarized in Table 1. The two cohorts did not differ significantly with respect to age, BMI, or surgical approach. In contrast, the two groups differed significantly with respect to smoking (Table 1), ASA score, the indication for surgery, the length of surgery, and the length of hospital stay.

As shown in Table 2, we found no significant difference between the two cohorts with respect to the total MED received. In contrast, we found significant differences in the distribution of opioids and NOAs administered intraoperatively and postoperatively. Specifically, we found that a significantly higher MED was administered intraoperatively in the Austrian cohort compared to the Canadian cohort (117.24 vs. 79.62, respectively; *p* < 0.001), whereas the women in the Austrian cohort received a significantly lower MED in the PACU compared to the Canadian cohort (25.96 vs. 30.42, respectively; *p* = 0.04). Nevertheless, pain level measured using a VAS before transfer from the PACU to the postoperative ward was lower in the Austrian compared to the Canadian cohort (1 vs. 3; *p* < 0.001, Table 1).

The primary outcome, MED within the first 24 h in the postoperative ward, was significantly lower in the Austria cohort compared to Canadian cohort (2.36 vs. 27.98, respectively; *p* < 0.001). To identify any potential predictive factors for MED administration in the first 24 h in the postoperative ward, we performed a regression analysis using the following variables: the use of intraoperative benzodiazepine, the intraoperative number of NOA doses administered, the intraoperative doses of co-analgesics, the number of NOA doses administered in the postoperative ward, the indication for surgery, the mode of hysterectomy (minimally invasive surgery vs. an open approach), total MED received intraoperatively and in the PACU, and country (Austria vs. Canada). As shown in Table 3, only two variables—country and mode of hysterectomy—were found to independently affect the MED received in the first 24 h after surgery.

At hospital discharge, 0/200 (0%) of the women in the Austrian cohort were given a prescription for opioids, compared to 197/200 (98.5%) of the women in the Canadian cohort (*p* < 0.001). However, the length of hospital stay was significantly shorter in Canada compared to Austria (1.5 vs. 5 days, respectively; *p* < 0.001, Table 1).

## 4. Discussion

The findings of this study indicate a difference in opioid-prescribing practices following hysterectomy, with a liberal practice in the tertiary care centre in Canada and a restrictive practice in the tertiary care centre in Austria. Although we found no significant difference with respect to the total amount of opioids administered in the first 24 h after surgery, the timing of administration (i.e., intraoperatively, in the PACU, and in the postoperative ward) differed markedly between the two cohorts.

A 2019 systematic review summarizing opioid-prescribing practices after minimally invasive hysterectomy reported a median MED of 32 (range: 14–74) within the first 24 h after surgery [18], which is comparable to the Canadian cohort, with a mean MED of 27.98 (SD, ±37.97). In contrast, women in the Austrian cohort received far fewer opioids in this same time period, with a mean MED of 2.36 (SD ± 7.50). In an attempt to explain these highly contrasting results, we performed a regression analysis using carefully selected and plausible confounding variables. An open approach versus a minimally invasive approach and the country in which the hysterectomy was performed were the only independent risk factors for higher MED administration in the postoperative ward.

In Austria, the multimodal opioid-sparing approach, based on the use and combination of various NOAs such as acetaminophen, metamizole, and diclofenac, is the first-line treatment for managing postoperative pain, consistent with the German Guideline for the Treatment of Acute Postoperative Pain and Posttraumatic Pain [19]. Opioids are used strictly as a rescue medication in cases in which NOAs alone do not achieve adequate pain relief. In contrast, in the tertiary care centre in Canada, postoperative pain was managed primarily using opioids, with NOAs used only to reduce the opioid dose, not primarily as a first-line treatment.

The growing opioid crisis in North America has been ascribed to a multitude of factors, including the commercialization of health care, aggressive marketing by pharmaceutical companies and prescribing physicians, and consumers’ socioeconomic and/or cultural attitudes towards pain and pain management [1,20]. In Austria, for example, patients often do not wish to receive opioid-based pain medications; in contrast, an observational study in the US found that patients were more satisfied with their care when they received an opioid prescription [21].

We found that the women in the Austrian cohort had a significantly longer hospital stay compared to the women in the Canadian cohort (5 days vs. 1.5 days, respectively), which is mainly attributed to Austrian hospital billing policies and tradition. As a consequence, 0% of patients in the Austrian cohort received an opioid prescription at discharge, compared to 98.5% of patients in the Canadian cohort. It is important to note that, independent of the length of stay, in the Austrian cohort, opioid administration was extremely rare within the first 24 h in the postoperative ward following surgery and during the entire hospital stay.

The strengths of this study include its comparison of two separate, well-matched cohorts, the prospective design of the Canadian cohort, and a relatively high sample size, particularly given the magnitude of the observed differences. On the other hand, this study has several limitations that warrant discussion. First, we studied only two tertiary care centres; thus, our results may not necessarily represent their entire respective countries or geographic regions. Second, additional risk factors for increased opioid consumption such as prior opioid use were not available for the Austrian cohort. Third, details regarding the regimen for the administration of medications such as PRN or at set times were not consistently available and therefore not analysed. Lastly, the mixed prospective and retrospective nature of the cohorts is a potential limitation. However, given the magnitude of the differences regarding opioid administration in the postoperative ward in the first 24 h after surgery (with a nearly 12-fold higher median MED administered in Canada compared to Austria), we do not believe that these factors affected our results in a meaningful way.

## 5. Conclusions

In conclusion, we found marked differences between two tertiary care centres in Austria and Canada with respect to their pain management strategies following elective hysterectomy for benign indications. Based on our findings, we hypothesize that besides an open vs. minimal invasive surgical approach, postoperative opioid administration in the hospital can be attributed—at least to a large extent—to cultural attitudes towards postoperative opioid administration. The significant differences in the perioperative distribution of NOAs might also be associated with loco-cultural pain management strategies and availabilities. The longer hospital stay in Austria appears to allow for the reduction—if not the complete abolishment—in opioid prescriptions at discharge following hysterectomy. Additional research is needed in order to explore how a culturally supported approach to reducing postoperative opioid administration and prescription can be achieved.

## Figures and Tables

**Table 1:** Patient characteristics of the Austrian (AUT) and Canadian (CAN) cohorts.

	AUT (*n* = 200)	CAN (*n* = 200)	*p*-Value
Age, years	49.9 ± 9.8	49.7 ± 9.4	0.82
BMI, kg/m^2^	26.8 ± 5.5	27.1 ± 6.8	0.19
Smoking, *n* (%)	51 (25.5%)	19 (9.5%)	<0.001
ASA classification, *n* (%)			<0.001
1	73 (36.5%)	33 (16.6%)	
2	110 (49.1%)	114 (57.3%)	
3	17 (8.5%)	50 (25.1%)	
4	0	2 (1%)	
Indication for surgery, *n* (%)			<0.001
Pain	4 (2%)	25 (12.5%)	
Other	196 (98%)	175 (87.5%)	
Mode of hysterectomy, *n* (%)			0.588
Minimally invasive	170 (85%)	165 (82.5%)	
Laparotomy	30 (15%)	35 (17.5%)	
VAS prior to PACU discharge	1 (0–6)	3 (0–9)	<0.001
Length of surgery, minutes	99.44 ± 41.35	109.66 ± 38.50	0.011
Length of hospital stay, days	5.01 ± 2.50	1.51 ± 1.27	<0.001

The data are presented as the mean ± standard deviation for numerical parameters, the median (range) for non-parametric variables, and a number (%) for categorical parameters. Minimally invasive includes laparoscopic, vaginal, robotic, and laparoscopic-assisted vaginal approaches. BMI, body mass index; ASA, American Society of Anesthesiologists; VAS, visual analogue scale; PACU, post-anaesthesia care unit.

**Table 2:** Perioperative and postoperative analgesic medications administered to the Austrian and Canadian cohorts.

	AUT	CAN	*p*-Value
	(*n* = 200)	(*n* = 200)	
Intraoperative			
MED	117.24 ± 53.80	79.62 ± 28.95	<0.001
Benzodiazepine	14 (7%)	131 (65%)	<0.001
NOA	0.37 (0.41)	1 (0.64)	<0.001
In the PACU			
MED	25.96 ± 18.61	30.42 ± 25.03	0.04
Total intraoperative and in the PACU			
MED	143.20 ± 56.24	110.04 ± 37.37	<0.001
First 24 h after leaving the PACU			
MED	2.36 ± 7.50	27.98 ± 37.97	<0.001
NOA	4.03 ± 2.1	3.57 ± 1.42	0.01
Total MED during hospital stay	145.59 ± 57.84	137.87 ± 55.02	0.17

The data are presented as the mean ± standard deviation or *n* (%). MED, morphine equivalent dose; NOA, non-opioid analgesic; PACU, post-anaesthesia care unit.

**Table 3:** The regression analysis of factors influencing the MED received in the first 24 h after leaving the PACU.

	Coefficient of Regression (Beta)	SE (β)	β	*p*-Value	95% CI
1 = Austria, 2 = Canada	23.52	3.81	0.39	<0.001	16.03–31.02
Intraoperative benzodiazepine	3.75	3.15	0.06	0.26	−2.62–9.75
Intraoperative additional NOA *	0.65	2.37	0.01	0.78	−4.00–5.31
Intraoperative additional co-analgesics ^†^	−1.10	1.93	−0.02	0.57	−4.88–2.70
Additional NOA in postoperative ward	0.14	0.71	0.01	0.84	−1.25–1.53
Mode of hysterectomy	−31.63	3.48	−0.39	<0.001	−38.46–−24.79
Indication for surgery	3.49	4.93	0.03	0.48	−6.19–13.18
Length of surgery	0.01	0.03	0.01	0.85	−0.06–0.07
Intraoperative and PACU MED	0.01	0.03	0.01	0.74	−0.04–0.06

* Including paracetamol, diclofenac, metamizole, and/or ketorolac. ^†^ Including clonidine, ketamine, lidocaine, and/or glucocorticoids. NOA, non-opioid analgesic; PACU, post-anaesthesia care unit; MED, morphine equivalent dose; CI, confidence interval.

## Data Availability

The original contributions presented in the study are included in the article, further inquiries can be directed to the corresponding author.

## References

[1] Ayoo K., Mikhaeil J., Huang A., Wasowicz M. (2020). The opioid crisis in North America: Facts and future lessons for Europe. Anaesthesiol. Intensive Ther..

[2] Young J.C., Wu J.M., Willis-Gray M., Pate V., Jonsson Funk M. (2020). Persistent Opioid Use After Hysterectomy in the United States, 2005–2015. Obs. Gynecol..

[3] Schieber L.Z., Guy G.P., Seth P., Young R., Mattson C.L., Mikosz C.A., Schieber R.A. (2019). Trends and Patterns of Geographic Variation in Opioid Prescribing Practices by State, United States, 2006–2017. JAMA Netw. Open.

[4] Thompson J.C., Komesu Y.M., Qeadan F., Jeppson P.C., Cichowski S.B., Rogers R.G., Mazurie A.J., Nestsiarovich A., Lambert C.G., Dunivan G.C. (2018). Trends in patient procurement of postoperative opioids and route of hysterectomy in the United States from 2004 through 2014. Am. J. Obs. Gynecol..

[5] Larach D.B., Sahara M.J., As-Sanie S., Moser S.E., Urquhart A.G., Lin J., Hassett A.L., Wakeford J.A., Clauw D.J., Waljee J.F. (2021). Patient Factors Associated with Opioid Consumption in the Month Following Major Surgery. Ann. Surg..

[6] Brunes M., Habel H., Altman D., Ek M. (2020). Risk-factors for continuous long-term use of prescription opioid drugs 3 years after hysterectomy: A nationwide cohort study. Acta Obs. Gynecol. Scand..

[7] Baruch A.D., Morgan D.M., Dalton V.K., Swenson C. (2018). Opioid Prescribing Patterns by Obstetrics and Gynecology Residents in the United States. Subst. Use Misuse.

[8] Edlund M.J., Martin B.C., Russo J.E., DeVries A., Braden J.B., Sullivan M.D. (2014). The role of opioid prescription in incident opioid abuse and dependence among individuals with chronic noncancer pain: The role of opioid prescription. Clin. J. Pain.

[9] Shah A., Hayes C.J., Martin B.C. (2017). Factors Influencing Long-Term Opioid Use among Opioid Naive Patients: An Examination of Initial Prescription Characteristics and Pain Etiologies. J. Pain.

[10] Shah A., Hayes C.J., Martin B.C. (2017). Characteristics of Initial Prescription Episodes and Likelihood of Long-Term Opioid Use—United States, 2006–2015. MMWR Morb. Mortal. Wkly. Rep..

[11] Centers for Disease Control and Prevention (2019). Opioid Overdose: Understanding the Epidemic. https://www.cdc.gov/overdose-prevention/about/understanding-the-opioid-overdose-epidemic.html.

[12] Levy B., Paulozzi L., Mack K.A., Jones C.M. (2015). Trends in Opioid Analgesic-Prescribing Rates by Specialty, U.S., 2007–2012. Am. J. Prev. Med..

[13] Fiore J.F., El-Kefraoui C., Chay M.A., Nguyen-Powanda P., Do U., Olleik G., Rajabiyazdi F., Kouyoumdjian A., Derksen A., Landry T. (2022). Opioid versus opioid-free analgesia after surgical discharge: A systematic review and meta-analysis of randomised trials. Lancet.

[14] van Amsterdam J., Pierce M., van den Brink W. (2021). Is Europe Facing an Emerging Opioid Crisis Comparable to the U.S.?. Ther. Drug Monit..

[15] McDonald D.C., Carlson K., Izrael D. (2012). Geographic variation in opioid prescribing in the U.S. J. Pain.

[16] Benlolo S., Hanlon J.G., Shirreff L., Lefebvre G., Husslein H., Shore E.M. (2021). Predictors of Persistent Postsurgical Pain After Hysterectomy-A Prospective Cohort Study. J. Minim. Invasive Gynecol..

[17] Luchting B., Rachinger-Adam B., Hulde N., Heyn J., Azad S.C. (2017). Challenging equipotency calculation for hydromorphone after long-term intravenous application. Ann. Palliat. Med..

[18] Johnson C.M., Makai G.E.H. (2019). A Systematic Review of Perioperative Opioid Management for Minimally Invasive Hysterectomy. J. Minim. Invasive Gynecol..

[19] DGAI AWMF-Leitlinie Behandlung Akuter Perioperativer und Postoperativer Schmerzen. https://www.awmf.org/uploads/tx_szleitlinien/001-025l_S3_Behandlung-akuter-perioperativer-posttraumatischer-Schmerzen_2022-03.pdf.

[20] Verhamme K.M.C., Bohnen A.M. (2019). Are we facing an opioid crisis in Europe?. Lancet Public Health.

[21] Jerant A., Agnoli A., Franks P. (2020). Satisfaction with Health Care Among Prescription Opioid Recipients. J. Am. Board Fam. Med..

